# Supplementary material for: The Construction and Exploration of a Comprehensive MicroRNA Centered Regulatory Network in Foxtail Millet (Setaria italica L.)
Source: Front Plant Sci. 2022 May 6;13:848474. doi: 10.3389/fpls.2022.848474 (PMC9121102; doi:10.3389/fpls.2022.848474)
Supplement: Supplementary file 5 [file Data_Sheet_5.zip › Sit-miR399b_Seita.9G218200.1_2155_TPlot.pdf]

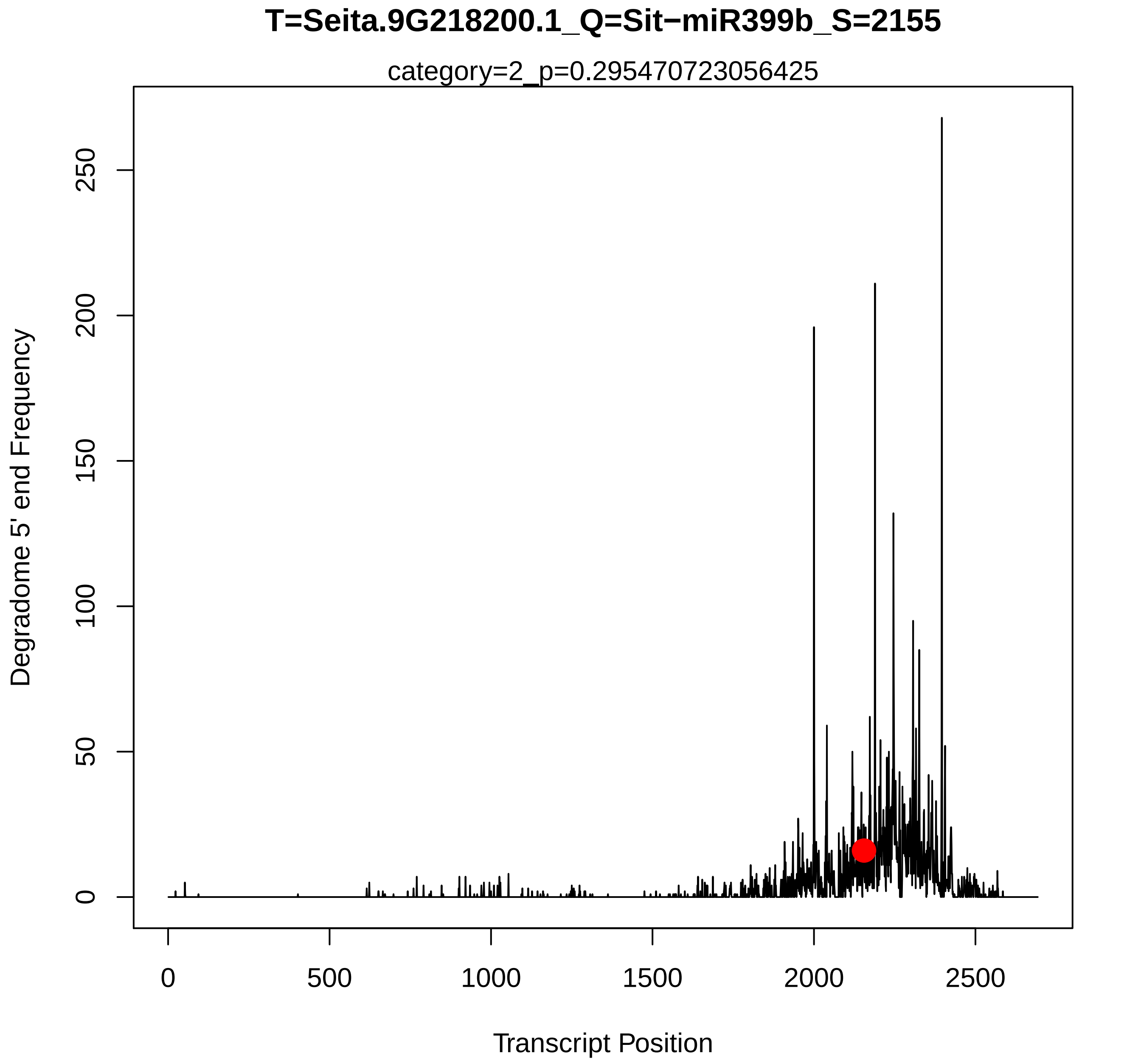

T=Seita.9G218200.1_Q=Sit−miR399b_S=2155
category=2_p=0.295470723056425
Degradome 5' end Frequency
Transcript Position
0
50
100
150
200
250
0
500
1000
1500
2000
2500
